# A quick relaxation exercise for people with chronic obstructive pulmonary disease: explorative randomized controlled trial

**DOI:** 10.1186/s40248-018-0124-9

**Published:** 2018-05-02

**Authors:** Eleonora Volpato, Paolo Banfi, Antonello Nicolini, Francesco Pagnini

**Affiliations:** 1 Department of Psychology, Università Cattolica del Sacro Cuore, Largo A. Gemelli, 1, Milan, Italy; 2 IRCCS Santa Maria Nascente, Fondazione Don Carlo Gnocchi, Milan, Italy; 3 Unità di Riabilitazione Respiratoria, ASL 4 Chiavarese, Ospedale di Sestri Levante, Sestri Levante, Italy; 4 Department of Psychology, Harvard University, Cambridge, MA USA

**Keywords:** Relaxation techniques, Quality of life, Pulmonary rehabilitation, Clinical psychology

## Abstract

**Background:**

People with Chronic Obstructive Pulmonary Disease (COPD) suffer from dyspnoea, which may be increased by anxiety. Previous studies suggest that relaxation techniques may have positive effects in pulmonary rehabilitation. The main aim of this study is to explore the clinical impact of a quick, one-session, relaxation training for people with COPD.

**Methods:**

In this perspective, 38 participants with COPD were recruited and randomly assigned to listen to a relaxing audio or to watch a neutral stimulus, during their routine exams. Participants were assessed for psychological and physiological variables, analysed through non-parametric tests.

**Results:**

Those who joined the relaxation training showed more positive outcomes about respiratory and cardiac assessments, as well as for state anxiety and positive affections, in comparison with the baseline and the control group.

**Conclusions:**

Study results suggest that relaxation has a potential to produce improvements in respiratory and cardiac functions, together with a positive emotional effect and a reduction of anxiety.

**Trial registration:**

ClinicalTrials.gov ID: NCT02698904. Record Registration: February 2016.

## Background

Chronic Obstructive Pulmonary Disease (COPD) is a complex, debilitating, and preventable lung condition, characterized by "persistent and progressive airflow limitation due to chronic inflammatory response in the airways and the lung to noxious particles or gases" with different degrees of impairment [1]. There is a progressive pulmonary function impairment that leads to a lower exercise tolerance and, in severe conditions, to the need for oxygen and possibly mechanical ventilation [2]. Other main symptoms of COPD include chronic cough, dyspnea, overproduction of sputum, wheezing, chest tightness, asthenia, weight loss, and anorexia [1]. All these symptoms have a negative impact on the quality of life [3, 4]. The prevalence of the diseases is increasing as there is an increasing exposure to risk factors [5–7]. In fact, it is estimated that COPD may become the third cause of death in the world by 2020 [8]. In this respect, in patients with advanced COPD and severe chronic hypoxemia refractory to maximal treatment with bronchodilator and anti-inflammatory drugs, smoking cessation and long-term oxygen therapy are the only two interventions that have unequivocally shown to reduce COPD mortality [1].

Pulmonary rehabilitation is very important at any stage of the disease, improving the ability to tolerate fatigue and dyspnea, with a beneficial effect on daily activities [9–12]. People with COPD are at risk of severe distress [13]. Depression and anxiety often appear together in these patients [14], even if the actual presence of psychological issues tends to be underestimated by the physicians [15]. Therefore, these aspects often remain untreated [14, 16, 17], with negative effects on the quality of life [18, 19].

Relaxation techniques are psychophysiological procedures that promote somatic and cognitive distension, relieving from tensions. These techniques are widely used in rehabilitation and are very effective against anxiety and distress [20, 21]. Both the physiological and the psychological effects of relaxation have a positive effect on people affected by pulmonary diseases [22], improving the oxygen saturation [23, 24] and promoting a reduction of anxiety and depression in people with COPD [25–27]. The cost-effectiveness of adding this technique to a rehabilitation program, however, is not clear [27, 28]. So far [29], relaxation training for people with COPD has often been tested in the mainframe of more general interventions (e.g., Cognitive Behavioral Therapy - CBT). We believe that a quick relaxation training, provided by an audio-guide right after their hospital exams, will promote positive emotions, reduce state anxiety and improve respiratory conditions. In this perspective, our aim is to investigate the feasibility of the relaxation technique’s application for people with COPD, exploring its preliminary efficacy and effectiveness in a short time of implementation. To date, no study has looked at verifying the experience of a brief relaxation exercise impact breathing and psychological parameters in people with COPD in the context of a clinical visit.

## Methods

### Study aims

The main aim of this study is to explore the effectiveness, feasibility and acceptability of a quick, one-session, relaxation training for people with Chronic Obstructive Pulmonary Disease (COPD) in a short time of implementation.

#### Design and setting of the study

We conducted an explorative, two-arms, single-blind Randomized Controlled Trial (RCT) to test the short-term effects of a one-session relaxation training based on mindful natural breathing on people with COPD. Thirty-eight subjects with COPD were recruited from the Respiratory Rehabilitation Unit of the Don Gnocchi Hospital, in Milan. The study was approved by the Don Gnocchi Foundation Ethical Committee. ClinicalTrials.gov ID: NCT02698904.

### Participants

Inclusion criteria were defined as follow: diagnosis of COPD confirmed by a specialized physician; age above or equal to 18 years; inpatients or outpatients; basal FEV_1_/FVC < 70% of reference values, using the standards established by Global Initiative for Chronic Obstructive Lung Disease (GOLD) [1]. Subjects were excluded in case of pregnancy, psychiatric disturbances, oncological diseases and comorbid states that determine an immunosuppressive condition.

**Fig. 1:**
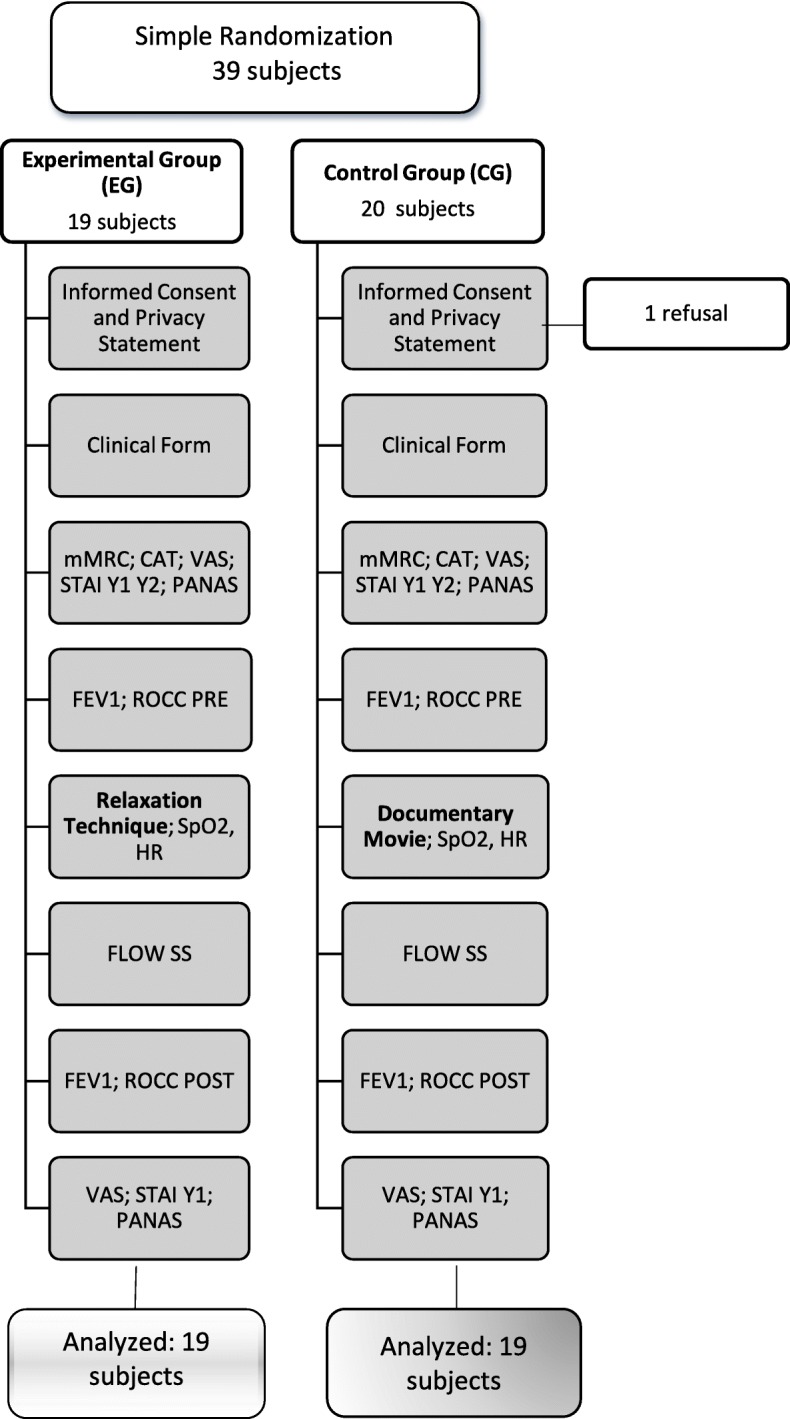
Flow Chart of the procedure

Subjects who met the inclusions criteria were randomly allocated to two groups. A simple randomization and an allocation ratio 1:1 were adopted (Fig. 1).

Informed consent was obtained. At baseline assessment, as well as at the end of the intervention (soon after), patients were assessed both about their psychological state (psychometric questionnaires) and their pulmonary function and physiological conditions (spirometry, saturation level and heart rate). While assessed, participants were blinded to their assignment.

#### Intervention

Subjects in the experimental group (EG) listened to a relaxing audio track of the duration of 11 min. The content of the audio track was a relaxation exercise based on the mindful natural breathing [30]. Instructions included becoming aware of the sensations of breathing, accepting them as they are at the present moment and non-judgmental awareness of mind wandering. Subjects laid down on a rigid but not hard bed, with a pillow, to facilitate muscle release, listening to the audio track with a pair of headphones. Instructions included focusing on the breathing mechanism, by purposefully paying attention to the breath, placing the left hand on the chest and the right on abdomen, improving the perception of torso movements. These exercises aim to increase the awareness of the respiratory process, leading to a relaxation response [31]. Relaxing music was played on the background. Subjects were advised to close their eyes.

Participants in the control group (CG), instead, watched a documentary movie that included neutral contents and the same duration of the relaxation audio, in a relaxing setting similar to the other group.

### Instruments

#### Physiological assessment

We assessed COPD severity with the lung function test, detecting Forced Vital Capacity (FVC), Forced Expiratory Volume in the First Second (FEV_1_), Tiffeneau-Pinelli Index (FEV_1_/FVC) with spirometer PONY FX (COSMED Srl, Rome, Italy). We also used the module Rocc Pony FX, which allows the measurement of airway resistance (kPa/l/s) using the technique of interrupter resistance (Rint). Furthermore, we detected the heart rate and oxygen saturation using pulse oximeter, Pulsox 300i (Konica Minolta, Inc.). The criteria used to determine if the spirometry tests were performed to an acceptable degree followed the Thoracic Society/European Respiratory Society Statement [32].

#### State trait anxiety inventory - Y1 and Y2 forms (STAI- Y1 Y2)

The State-Trait Anxiety Inventory (STAI) is a self-report assessment device which includes separate measures of state and trait anxiety [33].

#### Positive and negative affective schedule (PANAS) (state version)

The state version of Positive and Negative Affective Schedule (PANAS) was completed before and after treatment. It consists of 20 items, 10 for the scale of positive affect (PA) and 10 for the scale of negative affect (NA). The subject should evaluate his feelings with a five-point Likert scale. The original version was developed and validated by Watson, Clark and Tellegen in 1988 and it possesses excellent psychometric properties [34].

#### Visual analogue scales (VAS)

Patients scored their emotional state on a Visual Analogue Scale (VAS), before and after the treatment. In particular, each subject was asked to rate on a range of 0–7 the intensity of its liveliness, sadness, anger, surprise, anxiety, repugnance, fury and serenity at the moment of the administration.

#### Short flow state scale (short FSS-2)

A short form of Flow State Scale, called Short FSS-2, was completed after treatment to assess the possible state of flow experience [35].

### Statistical analysis

Data analysis was conducted with the statistical software Statistical Package for Social Science (SPSS). Given the relatively small sample and the expectation of a non-normal distribution, we opted for the use of non-parametric tests. Differences between the two groups were analyzed with the Mann-Whitney U test, while within analyses were conducted with the Wilcoxon test. The significance level was set to 0,05.

## Results

### Sample characteristics

The study sample (see Table 1) was composed by 38 participants (23 males and 15 females), with a mean age of 72.66 years old. The mean length of illness was 2.03 years. All subjects had moderately severe chronic airflow limitations with an average FEV_1_ of 54.26% of the reference value and an average FVC of 72.11%.

**Table 1:** Comparison of socio-demographic characteristics and baseline parameters of patients in overall sample, study and control groups (*p* < 0.05)^a^

Variables	Experimental group (*n* = 19)	Control group (*n* = 19)
Socio- demographic data		
Sex (*n*, %)		
Female	8 (42.10)	7 (36.80)
Male	11 (57.90)	12 (63.20)
Age (years) (Mean, SD)	72.53 (10.30)	72.79 (7.06)
Weight (Mean, SD)	72.32 (16.63)	69.59 (13.24)
Height (Mean, SD)	164.32 (8480)	165.26 (10.13)
BMI^b^ (Mean, SD)	26.88 (6.46)	25.5 (4.85)
Educational level (*n*, %)		
None	1 (5.30)	0 (0)
Primary School	7 (36.80)	9 (47.40)
Junior High School	7 (36.80)	6 (31.60)
High School	4 (21.10)	3 (15.80)
Graduated or higher education	0 (0)	1 (5.30)
Profession/Job done longer in the past (*n*, %)		
Housewife	4 (21.10)	2 (10.50)
Employee	5 (26.30)	2 (10.50)
Laborer	4 (21.05)	8 (42.10)
Professional	5 (26.30)	5 (26.30)
Manager	1 (5.30)	2 (10.50)
Medical and clinical data		
Duration of Illness (Mean, SD)	1.79 (0.78)	2.26 (0.80)
5 (*n*, %)	8 (42.10)	4 (21.10)
10 (*n*, %)	7 (36.80)	6 (31.60)
> 15 (*n*, %)	4 (21.10)	9 (47.40)
Smoking (*n*, %)		
Never	2 (10.50)	0 (0)
Yes	6 (31.10)	7 (36.80)
Quitted	11 (57.90)	12 (63.20)
Drugs (*n*, %)		
LAMA I^b^	15 (78.90)	17 (89.50)
LABA I^b^	17 (89.5)	18 (94.70)
ICS^b^	12 (63.20)	10 (52.60)
Anxiolytics	3 (15.80)	9 (47.40)
Antidepressants	3 (15.80)	4 (21.10)
Exacerbation, last year (*n*, %)		
< 1	12 (63.20)	10 (52.60)
1–3	6 (31.60)	9 (47.40)
> 3	1 (5.30)	0 (0)
Hospitalization, last year (*n*, %)		
< 1	13 (68.40)	11 (57.90)
2	5 (26.30)	8 (42.10)
> 2	1 (5.30)	0 (0)
mMRC^c^	2.47 (0.51)	2.21 (0.41)
CAT^c^	28.79	30.68

^a^Data are presented as Mean+/-Standard Deviation (SD) and number, percentage (*n*, %)

^b^*BMI* Body Mass Index, *LAMA* Long-Acting Muscarinic Antagonists, *LABA* Long-Acting Beta 2-Adrenergic Agonists, *ICS* Inhaled Corticosteroids

^c^*mMRC* Modified Medical Research Council Dyspnoea Scale, *CAT* COPD Assessment Test

### Differences between groups before and after treatment

After the treatment, we found significant differences (Table 2) in favor of the EG in comparison with the CG concerning heart rate (U = 95, *z* = − 2504, *p* = 0.006). There were also significant differences between groups about both emotional states, assessed by VAS (U = 108, *z* = − 2129, *p* = 0.016), and anxiety state, detected by STAI-Y1 (U = 104.5, *z* = 2223, *p* = 0.013).

**Table 2:** Comparison of psychological characteristics, pulmonary functions and other clinical data between groups at the end of the study (*p* < 0.05)^a^

	Baseline			Post - treatment		
Variables	Experimental Group (EG; *n* = 19)	Control Group (CG; *n* = 19)	Mann-Whitney Test *p (U, Z)*	Experimental Group (EG; *n* = 19)	Control Group (CG; *n* = 19)	Mann-Whitney Test *p (U, Z)*
Psychological states						
STAI-Y1^b^ (Mean +/− SD)	33.68 (6.48)	36.68 (10.26)	U = 151.5 *z* = − 0.848 *p* = 0.198	26.05 (5.04)	31.26 (7.43)	U = 104.5 *z* = − 2223 *p* = 0.013*
PANAS^b^ (Mean +/− SD)						
Positive	2.92 (0.24)	3.05 (0.46)	U = 159 *z* = − 0.633 *p* = 0.2635	3.17 (0.36)	3.01 (0.38)	U = 139.5 *z* = − 1202 *p* = 0.114
Negative	1.39 (0.48)	1.45 (0.43)	U = 155 *z* = − 0.751 *p* = 0.226	1.12 (0.26)	1.12 (0.20)	U = 161 *z* = − 0.638 *p* = 0.261
VAS^b^ (Mean +/-SD)	20.53 (5.07)	20.68 (5.45)	U = 173.5 *z* = − 0.206 *p* = 0.418	19.79 (3.76)	17.68 (3.95)	U = 108 *z* = − 2129 *p* = 0.016*
FSS-2^b^ (Mean +/-SD)				33.63 (3.74)	33.57 (3.06)	U = 174 *z* = − 0.191 *p* = 0.424
Psychological traits						
STAI-Y2^b^ (Mean+/− SD)	36.63 (11.96)	39 (11.04)	U = 155 *z* = − 0.745 *p* = 0.228			
Pulmonary functions and physiological data						
FEV_1_^b^ (Mean +/-SD)	1.35 (0.74)	1.24 (0.51)	U = 175 *z* = − 0.161 *p* = 0.436	1.39 (0.75)	1.26 (0.47)	U = 168.5 *z* = − 0.350 *p* = 0.363
FVC^b^ (Mean +/-SD)	2.14 (0.94)	2.27 (0.89)	U = 167 *z* = − 0.394 *p* = 0.346	1.97 (0.93)	2.38 (0.81)	U = 127 *z* = − 1562 *p* = 0.059
ROCC^b^, kPa/l/s (Mean +/-SD)	0.34 (0.14)	0.30 (0.12)	U = 151.5 *z* = − 0.847 *p* = 0.198	0.40 (0.19)	0.30 (0.15)	U = 135.5 *z* = − 1314 *p* = 0.094
SpO_2_^b^ (Mean +/-SD)	91.63 (3.18)	95.58 (2.79)	U = 110.5 *z* = − 2058 *p* = 0.020*	93.84 (2.73)	94 (2.62)	U = 175.5 *z* = − 0.148 *p* = 0.441
HR^b^ (Mean+/-SD)	69.32 (8.80)	73.95 (11.47)	U = 140.5 *z* = − 1171 *p* = 0.121	65.47 (7.87)	73.05 (11.75)	U = 95 *z* = − 2504 *p* = 0.006*

^a^Data are presented as Mean+/-Standard Deviation (SD) and number, percentage (*n*, %)

^b^*STAI-Y1* State Trait Anxiety Inventory-State Form, *PANAS* Positive and Negative Affective Schedule-State Form, *VAS* Visual Analogue Scale, *FSS-2* Short Flow State Scale 2, *STAI-Y2* State Trait Anxiety Inventory-Trait Form, *FEV*_*1*_ Forced Expiratory Volume in the First Second, *FVC* Forced Vital Capacity, ROCC, *kPa/l/s* Measurement of airway resistance, *SpO*_*2*_ Oxygen saturation, *HR* Heart Rate

### Analysis of the variables within groups before and after treatment

Comparing pre-post intervention (Table 3), the detection of respiratory resistance allowed to detect notable differences only within the experimental group (*z* = − 1851 *p* = 0.032). Similarly, the heart rate had an important decrease in the EG (*z* = − 3576, *p* = 0.000) and not in the CG (*z* = − 1776, *p* = 0.038).

**Table 3:** Comparison of psychological characteristics, pulmonary functions and other clinical data at baseline and after treatments of patients both in study group and control group (*p* < 0.05)^a^

	Experimental group (EG; *n* = 19)			Control group (CG; *n* = 19)		
Variables	Baseline	Post - treatment	Wilcoxon Test *z, p*	Baseline	Post - treatment	Wilcoxon Test *z, p*
Psychological states						
STAI-Y1^b^ (Mean +/− SD)	33.68 (6.48)	26.05 (5.04)	*z* = − 3771 *p* = 0.000*	36.68 (10.26)	31.26 (7.43)	*z* = − 3010 *p* = 0.001*
PANAS^b^ (Mean +/− SD)						
Positive	2926 (0.24)	3.17 (0.36)	*z* = − 2581 *p* = 0.005*	3.05 (0.46)	3.01 (0.38)	*z* = − 0.88 *p* = 0.465
Negative	1395 (0.48)	1.12 (0.26)	*z* = − 3127 *p* = 0.001*	1.45 (0.43)	1.12 (0.20)	*z* = − 3186 *p* = 0.000*
VAS^b^ (Mean +/-SD)	20.53 (5.07)	19.79 (3.76)	*z* = − 0.284 *p* = 0.388	20.68 (5.45)	17.68 (3.95)	*z* = − 2605 *p* = 0.004*
Psychological traits						
STAI-Y2^b^ (Mean+/− SD)	36.63 (11.96)			39 (11.04)		
Pulmonary functions and physiological data						
FEV_1_^b^ (Mean +/-SD)	1.35 (0.74)	1.39 (0.75)	*z* = − 0.735 *p* = 0.231	1.24 (0.51)	1.26 (0.47)	*z* = − 1702 *p* = 0.044*
FVC^b^ (Mean +/-SD)	2143 (0.94)	1.97 (0.93)	*z* = − 2378 *p* = 0.008*	2.27 (0.89)	2.38 (0.81)	*z* = − 2335 *p* = 0.010*
ROCC^b^, kPa/l/s (Mean +/-SD)	0.34 (0.14)	0.40 (0.19)	*z* = − 1851 *p* = 0.032*	0.30 (0.12)	0.30 (0.15)	*z* = − 0.362 *p* = 0.358
vSpO_2_^b^ (Mean +/-SD)	91.63 (3.18)	93.84 (2.73)	*z* = − 2672 *p* = 0.004*	95.58 (2.79)	94 (2.62)	*z* = − 1327 *p* = 0.092
HR^b^ (Mean+/-SD)	69.32 (8.80)	65.47 (7.87)	*z* = *−* 3576 *p* = 0.000*	73.95 (11.47)	73.05 (11.75)	*z* = − 1776 *p* = 0.038*

^a^Data are presented as Mean+/-Standard Deviation (SD) and number, percentage (*n*, %)

^b^*STAI-Y1* State Trait Anxiety Inventory-State Form, *PANAS* Positive and Negative Affective Schedule-State Form, *VAS* Visual Analogue Scale, *FSS-2* Short Flow State Scale 2, *STAI-Y2* State Trait Anxiety Inventory-Trait Form, *FEV*_*1*_ Forced Expiratory Volume in the First Second, *FVC* Forced Vital Capacity, ROCC, *kPa/l/s* Measurement of airway resistance, *SpO*_*2*_ Oxygen saturation, *HR* Heart Rate

Regarding the psychological states, there was a significant change about the positive affects only within the experimental group (*z* = − 2581, *p* = 0.005); while there was a significance change about the negative affections in both groups (experimental group: *z* = − 3127, *p* = 0.001; control group: *z* = − 3186, *p* = 0.000).

## Discussion

We conducted a Randomized Control Trial to evaluate the impact of a relaxation technique on psychological and physiological well-being of people with COPD. The findings of this study suggest that a brief relaxation technique, based on mindful natural breathing, can be a helpful tool to improve psychological well-being and symptoms management of people with COPD. In particular, this mindful natural breathing improved the blood oxygenation load and FVC, as well as breathing resistance. That means, that respiratory levels improved after an 11-min guided relaxation training. This result supports the hypothesis that relaxation techniques can promote better blood oxygenation, in line with previous studies [23, 36]. It is also reported a significant decrease in heart rate: this other data corroborates the results of previous works on relaxation techniques that have found a decrease in heart beats per minute [37].

The relaxation audio had a positive emotional effect, increasing positive affects and reducing negative affections, confirming the hypothesis and previously obtained results [37, 38]. Furthermore, anxiety was significantly reduced after the intervention, both in comparison with the baseline assessment and with the control group. This confirms the idea that relaxation is one of the main treatments for anxiety [39, 40].

Study results could be useful in the clinical practice and highly reproducible. The intervention was held in a single session and with the use of an audio-recorded relaxing track. Furthermore, this type of quick relaxation exercise could be useful during daytime activities of people with COPD, especially when they are alone or when anxiety emerges as a possible attack of dyspnea. Relaxation techniques can, in fact, be practiced at any time, even on their own. Furthermore, relaxation techniques can be incorporated into other interventions, helping to reduce anxiety and depression. Therefore, the inclusion of relaxation training in the clinical routine of pulmonary services with COPD could be easily accomplished and could provide positive effects. In fact, even if our study was limited by a single session of the intervention, it was able to alleviate anxiety and improve well-being, with a highly scalable approach. Nevertheless, it is also limited by its relatively small sample size. Furthermore, generalization of the results from this study is restricted by the participants’ characteristics, who represented a narrow range of education levels, with basically no subjects with a graduate degree. The study was conducted in single-blind mode, therefore the researcher was aware of the group of the subject. Even if self-report and physiological assessments may be less influenced than other instruments, we recognize that limitation. Furthermore, it is possible to note that the main differences between groups are limited to the anxiety state and the dyspnea perceived as well as the heart rate, confirming that they are intrinsically related: indeed, sympathetic arousal is a physiological manifestation of anxiety. This result could also suggest the importance to improve the patient’s education in practicing the technique in order to obtain more significant short-term effects. Finally, the study was conducted in the hospital, during regular exams, and therefore it is not generalizable outside this setting.

Future works may consider a larger and more representative sample, with assessments from multiple settings. Nonetheless, further studies are needed to investigate better the efficacy and effectiveness of a relaxation technique based on mindful natural breathing, taking advantages of more than one session, because a long-term practice could boost beyond the benefits.

Motivation and concentration are essential in the practice of relaxation. These two elements require a certain level of patients’ engagement, that can be stimulated by health practitioners. Our findings indicate that relaxation techniques could pay an important role in intervention strategies, contributing to an improvement of the QoL and the well-being of people with COPD. The possible impact of this use of relaxation training on health costs may deserve a future investigation.

## Conclusions

The findings of our study show that a relaxation technique based on mindful natural breathing might have a potential to improve the COPD patients’ overall well-being. In particular, mindful natural breathing induces significant improvements in respiratory and cardiac functions, together with a positive emotional effect and a reduction of anxiety. Although the present study is a short-term one and its conclusions must be confirmed by further studies, it was required before a long-term investigation, in order to examine the endurance and the utility of the treatment to the patients.

## Abbreviations

CBT: Cognitive Behavioral Therapy
CG: control group
COPD: Chronic Obstructive Pulmonary Disease
EG: experimental group
FEV_1_: Forced Expiratory Volume in the First Second
FEV_1_/FVC: Tiffeneau-Pinelli Index
FVC: Forced Vital Capacity
GOLD: Global Initiative for Chronic Obstructive Lung Disease
NA: negative affect
PA: positive affect
PANAS: Positive and Negative Affective Schedule
RCT: Randomized Control Trial
Short FSS-2: Short Flow State Scale
SPSS: Statistical Package for Social Science
STAI: State-Trait Anxiety Inventory
VAS: Visual Analogue Scales
